# Replacement of the Alpha variant of SARS-CoV-2 by the Delta variant in Lebanon between April and June 2021

**DOI:** 10.1099/mgen.0.000838

**Published:** 2022-07-25

**Authors:** Georgi Merhi, Alexander J. Trotter, Leonardo de Oliveira Martins, Jad Koweyes, Thanh Le-Viet, Hala Abou Naja, Mona Al Buaini, Sophie J. Prosolek, Nabil-Fareed Alikhan, Martin Lott, Tatiana Tohmeh, Bassam Badran, Orla J. Jupp, Sarah Gardner, Matthew W. Felgate, Kate A. Makin, Janine M. Wilkinson, Rachael Stanley, Abdul K. Sesay, Mark A. Webber, Rose K. Davidson, Nada Ghosn, Mark Pallen, Hamad Hasan, Andrew J. Page, Sima Tokajian

**Affiliations:** ^1^​ Department of Natural Sciences, Lebanese American University, School of Arts and Sciences, Byblos, Lebanon; ^2^​ Quadram Institute Bioscience, Norwich Research Park, Norwich, Norfolk, UK; ^3^​ Ministry of Public Health, Epidemiological Surveillance Program, Museum Square, Beirut, Lebanon; ^4^​ National Influenza Centre Research Laboratory, Rafic Hariri University Hospital, Beirut, Lebanon; ^5^​ Laboratory of Molecular Biology and Cancer Immunology, Faculty of Sciences, Lebanese University, Lebanon; ^6^​ University of East Anglia, Norwich, Norfolk, UK; ^7^​ Norfolk and Norwich University Hospital, Norwich, Norfolk, UK; ^8^​ MRC Unit The Gambia at LHSTM, Fajara, Gambia; ^9^​ Ministry of Public Health, Beirut, Lebanon

**Keywords:** SARS-CoV-2, sequencing, genomic epidemiology, bioinformatics

## Abstract

The COVID-19 pandemic continues to expand globally, with case numbers rising in many areas of the world, including the Eastern Mediterranean Region. Lebanon experienced its largest wave of COVID-19 infections from January to April 2021. Limited genomic surveillance was undertaken, with just 26 SARS-CoV-2 genomes available for this period, nine of which were from travellers from Lebanon detected by other countries. Additional genome sequencing is thus needed to allow surveillance of variants in circulation. In total, 905 SARS-CoV-2 genomes were sequenced using the ARTIC protocol. The genomes were derived from SARS-CoV-2-positive samples, selected retrospectively from the sentinel COVID-19 surveillance network, to capture diversity of location, sampling time, sex, nationality and age. Although 16 PANGO lineages were circulating in Lebanon in January 2021, by February there were just four, with the Alpha variant accounting for 97 % of samples. In the following 2 months, all samples contained the Alpha variant. However, this had changed dramatically by June and July 2021, when all samples belonged to the Delta variant. This study documents a ten-fold increase in the number of SARS-CoV-2 genomes available from Lebanon. The Alpha variant, first detected in the UK, rapidly swept through Lebanon, causing the country's largest wave to date, which peaked in January 2021. The Alpha variant was introduced to Lebanon multiple times despite travel restrictions, but the source of these introductions remains uncertain. The Delta variant was detected in Gambia in travellers from Lebanon in mid-May, suggesting community transmission in Lebanon several weeks before this variant was detected in the country. Prospective sequencing in June/July 2021 showed that the Delta variant had completely replaced the Alpha variant in under 6 weeks.

## Data Summary

Consensus genomes are available from GISAID [1], the primary global repository for SARS-CoV-2 genomic data, subject to minimum quality control criteria. Raw reads and consensus genomes are available without restriction from the European Nucleotide Archive (ENA) with the data mirrored to the NCBI and DDBJ under Bioproject PRJEB46859. Accession numbers for GISAID and the ENA, metrics and de-identified case metadata are available for each sample in Table S1 (available in the online version of this article).

**Impact Statement**

The COVID-19 pandemic is a global problem, but only a small proportion of genomic surveillance has been undertaken in low- and middle-income countries, despite these countries containing most of the world’s population. Without genomic epidemiology data for a country, policy-makers and public health officials are hindered. We have undertaken a retrospective study looking at samples from Lebanon over a 5 month period, and showed different dynamics to those of other countries, such as the UK. Travel restrictions did appear to work to stem the flow of the Alpha variant from the UK. New variants of concern were also circulating in the community long before they were officially detected. This demonstrates the utility of genomic surveillance and highlights that different countries face different dynamics and challenges.

## Introduction

Since the first report from Wuhan, China, in December 2019 [2], COVID-19 has spread globally. The causative agent, severe acute respiratory syndrome-related coronavirus 2 (SARS-CoV-2), has caused (as of 31 July 2021) more than 200 million cases and more than four million deaths worldwide [3] and more than 562 527 cases and 7 909 deaths in Lebanon (https://www.moph.gov.lb/en/). After a period of relative stasis, since late 2020 the evolution of SARS-CoV-2 has been linked to Variants of Concern (VOCs) characterized by increased epidemic potential [4] and a distinctive set of mutations on the sequenced genomes. VOCs have been assigned letters of the Greek alphabet in the WHO naming scheme [5]. Of these, the Alpha variant (PANGO lineage B.1.1.7) [6, 7] was first detected in the UK in September 2020, while the Delta variant (PANGO lineage B.1.617.2) was first detected in India in October 2020 [8]. The Alpha variant had a 1.6× greater transmissibility over wildtype SARS-CoV-2 [9] with the N501Y mutation in the spike protein being linked to increased binding affinity in the ACE2 receptor [6]. The Delta variant had a an around 1.5× greater transmissibility than the Alpha variant [10] with multiple mutations in the spike protein which were linked to increased transmissibility and which arose multiple times independently, such as P681R and L452R which are linked to ACE2 receptor binding ability [11].

The first case of COVID-19 was reported in Lebanon on 21 February 2020. From March to July 2020, the number of cases remained low, with fewer than 100 cases daily. Subsequently, case numbers increased during a time of major policy changes and societal challenges: the reopening of Beirut airport to international travellers; an explosion at Beirut Port on 4 August —one of the largest non-nuclear explosions ever recorded [12] – displacing much of the population of the city; and the easing of the lockdown restrictions, leading by December 2020 to record highs (over 1000) in the number of COVID-19 cases per day [3].

The first case of the Alpha variant in Lebanon was detected on 22 December 20202 in a traveller arriving from the UK. This was followed by a surge in cases, triggering increased containment measures and a vaccination campaign. As a result, by early June 2021 rates had returned to low levels [13]. A subsequent easing of restrictions led to the return of many Lebanese expatriates and by the end of June 2021, case numbers had increased.

Sequencing of SAR-CoV-2 genomes from Lebanese samples has proven challenging because: reductions in air traffic have caused disruptions to supply chains, particularly those including a cold chain; increased demand for consumables in high-income countries has led to marked and prolonged shortages in low- and middle-income countries; and the high per-sample costs of sequencing.

With the emergence of multiple VOCs, the WHO has encouraged genomic surveillance worldwide. Rapid genome sequencing has been used widely in public health, in investigation of outbreaks [14], identification of novel VOCs [15, 16], and tracking the source and spread of variants [17].

Limited genomic surveillance was undertaken in the Lebanon in early 2021, with just 26 SARS-CoV-2 genomes available for the study period, nine of which were from travellers from Lebanon detected by other countries. Additional genome sequencing was thus needed to allow surveillance of variants in circulation. In total, 905 SARS-CoV-2 genomes were sequenced, representing a ten-fold increase in the number of genomes available for the country. The genomes were derived from SARS-CoV-2-positive samples, selected retrospectively from the sentinel COVID-19 surveillance network, to capture diversity of location, sampling time, sex, nationality and age. Although 16 PANGO lineages were circulating in Lebanon in January 2021, by February there were just four, with the Alpha variant accounting for 97 % of samples. In the following 2 months, all samples contained the Alpha variant. However, this had changed dramatically by June and July, when all samples belonged to the Delta variant. The Alpha variant, first detected in the UK, rapidly swept through Lebanon, causing the country’s largest wave to date, which peaked in January 2021. The Alpha variant was introduced to Lebanon multiple times despite travel restrictions, but the source of these introductions remains uncertain. The Delta variant was detected in Gambia in travellers from Lebanon in mid-May, suggesting community transmission in Lebanon several weeks before this variant was detected in the country. Prospective sequencing in June/July 2021 showed that the Delta variant had completely replaced the Alpha variant in under 6 weeks.

Without genomic epidemiology data for a country, policy-makers and public health officials are hindered. This study reveals slightly different dynamics to those of other countries, such as the UK. Travel restrictions did appear to work to stem the flow of the Alpha variant from the UK. New VOCs were also circulating in the community long before they were officially detected. This demonstrates the utility of genomic surveillance and highlights that different countries face different dynamics and challenges.

## Methods

### Samples

#### Sentinel surveillance

The sentinel surveillance system for influenza and COVID-19 like illness was implemented in Lebanon in late 2020 with the objective to monitor circulation of influenza and SARS-CoV-2 viruses. The network includes 18 outpatient sites such as primary health care centers, hospital emergency departments, and clinics in camps for refugees and displaced populations. The sites cover 7 of the 8 Lebanese provinces. In each site, one day per week is dedicated for the surveillance where any patient fitting the WHO case definitions for Influenza or COVID-19 like illness, is included. On site, a trained team is responsible for data collection (variables related to identification, demographic, clinical and exposure) and sample collection (nasopharyngeal swab in mainly viral transport media/ viral stabilizer media). Clinical samples are referred on the same working day to the National Influenza Center (NIC) where RT-PCR is conducted for SARS-CoV-2. Positive samples are kept at NIC for potential genomic analysis, and all samples sequenced as part of this study were from this sentinel surveillance dataset. Positive cases are integrated into the national COVID-19 surveillance database.

#### Sample description

For this study a total of 905 SARS-CoV-2 positive samples were collected from Lebanon, selected at random from clinical diagnostic samples, to provide a representative view of regions within Lebanon as shown in Fig. S1. These were collected through the sentinel surveillance system described previously. The samples represented a broad range of ages and sexes (Fig. S2) and were collected from a wide range of nationalities (Fig. S3). All cases were symptomatic at time of sample collection.

Samples with a diagnostic polymerase chain reaction (PCR) cutoff value of less than 25 cycles were selected to maximise the chance of yielding a high quality genome. Of the 8 provinces, 2 (Beirut and Akkar) were underrepresented compared to their populations. Samples were selected with low Ct levels, from different families and sites, regardless of nationality.

The samples were collected from 2020-12-29 to 2021-05-04. Of these 96.68 % (n=873 produced genomes of sufficient quality to allow for PANGO lineages to be called. Additionally, all samples (n=77) deposited in the GISAID SARS-CoV-2 public database and available on 2021-08-03 were added if they were noted as being from Lebanon, or from travellers from Lebanon. In particular, there were 20 additional sequences labelled Lebanon in GISAID that were known to be from travellers from outside Lebanon, which were removed from the analysis as they were exposed in another country.

### Genome sequencing and analysis

Samples were transported to the UK for high throughput genome sequencing. Primary samples were inactivated at the Department of Microbiology at the Norfolk and Norwich University Hospital Foundation Trust and extracted at the Bob Champion Research and Education laboratory at the University of East Anglia. Viral RNA was extracted using the Thermo Fisher Scientific MagMAX™ Viral/Pathogen II (MVP II) Nucleic Acid Isolation CE-IVD kit where 265 μl of Binding Buffer was added to 200 μl of the primary sample in a 96-deepwell plate and incubated at room temperature for 15 mins to inactivate the sample. Following sample inactivation 5 μl of Proteinase K and 10 μl of Binding Bead Mix were added. The remaining extraction steps were performed using the Thermo Scientific Flex System and the MVP_2wash_200_flex program using 500 μl of wash buffer, 500 μl 80 % ethanol followed by elution of RNA in 50 μl molecular grade, RNAse free water. Viral RNA was converted in cDNA and was then amplified using the ARTIC protocol v3 (LoCost) [18] with sequencing libraries prepared using CoronaHiT [19]. Genome sequencing was performed using the Illumina NextSeq 500 platform with one positive control and one negative control per 94 samples.

The raw reads were demultiplexed using bcl2fastq (v2.20). The reads were used to generate a consensus sequence using the ARTIC bioinformatic pipeline [20]. Briefly, the reads had adapters trimmed with TrimGalore [21] and were aligned to the Wuhan Hu-1 reference genome (accession MN908947.3) using BWA-MEM (v0.7.17) [22]; the ARTIC amplicons were trimmed and a consensus built using iVAR (v.1.3.0) [23]. PANGO lineages assigned using Pangolin (v3.1.7) and PangoLEARN model dated 2021-07-28 [24].

### Phylogenetic and clustering analysis

For the phylogenetic analysis, all sequences from GISAID where Lebanon is the country of exposure (Table S2) were downloaded and added to the sequences from the current study. All remaining sequences from GISAID were then compared to this data set where we kept the four closest ones for subsequent analysis to provide context. The alignment and neighbour search were done with uvaia (https://github.com/quadram-institutebioscience/uvaia), and problematic (homoplasic or difficult to sequence) sites were masked from the alignment [25]. Sequences with more than 50 % N were excluded from analysis, and a maximum likelihood tree was estimated with IQTREE2 [26] under an HKY model [27] with gamma rate heterogeneity [28]. For sequences with complete date information, we estimated divergence times using treetime using an autocorrelated molecular clock under the maximum likelihood tree. In particular, we used the autocorrelated clock model to estimate branch-wise substitution rates for all sequences, and then we removed the outlier sequences, from the 5 % higher percentile of evolutionary rates. We then estimated divergence times from the remaining samples assuming an uncorrelated relaxed clock model with a mean clock rate of 8x10-4 substitutions per site per year. This model allows for the individual rates to vary between samples, while the particular mean rate chosen reflects the timespan of the analysis [29]. Variants of concern are associated with episodic increases in the evolutionary rate, however sustained changes in the overall rate haven‟t been observed so far [30].

The number of transmission events into or outside Lebanon was found by ancestral reconstruction of nodes using castor for R [31], which calculates the probability of each ancestral node belonging to Lebanon or not based on the country of exposure information at the tips. Importation events are then the nodes with P (Lebanon) <50 % with at least one child node with P (Lebanon) = 100 %, and exportation nodes are equivalently nodes with P (Lebanon) >50 % with one or more nodes where P (Lebanon)=0 %. The exposure information comes from the “Location” information on the GISAID metadata, and may contain errors (in case the data providers cannot ascertain the travel history of samples).

Besides the phylogenetic analysis above, we used uvaia on a broader search, to find closest matches over different countries. Here, we used uvaia to compare all Alpha samples from Lebanon to the remaining sequences from GISAID, and kept the 128 closest neighbours per sample. In total 5 208 distinct global sequences were included. Since the same country may be represented more than once amongst these 128 neighbours, the best match per country is the sample with the highest number of perfect matches for each country. Not all countries are represented in all samples, and a “perfect match” considers only the four bases ACGT (any other disagreement is a mismatch, where only sites excluding Ns are considered). For this analysis we did not mask any sites in the alignment.

### Mutation Analysis

The NextStrain [22] open dataset (version 2022-03-09), based on genomes deposited with INSDC public databases [32], consisting of 4 119 969 genomes, was analysed to understand frequencies of mutations present in Lebanon with respect to the global frequencies, for each of the major variants of concern, Alpha, Delta and Omicron. This dataset was chosen as the data is openly available without restriction, unlike GISAID which places onerous constraints on data sharing and reuse. This can show if mutations that emerged regionally then independently emerged in later variants of concern at a higher level, indicating a mutation which may be linked to increased fitness.

## Results

### Establishment of Alpha

**Fig. 1.**
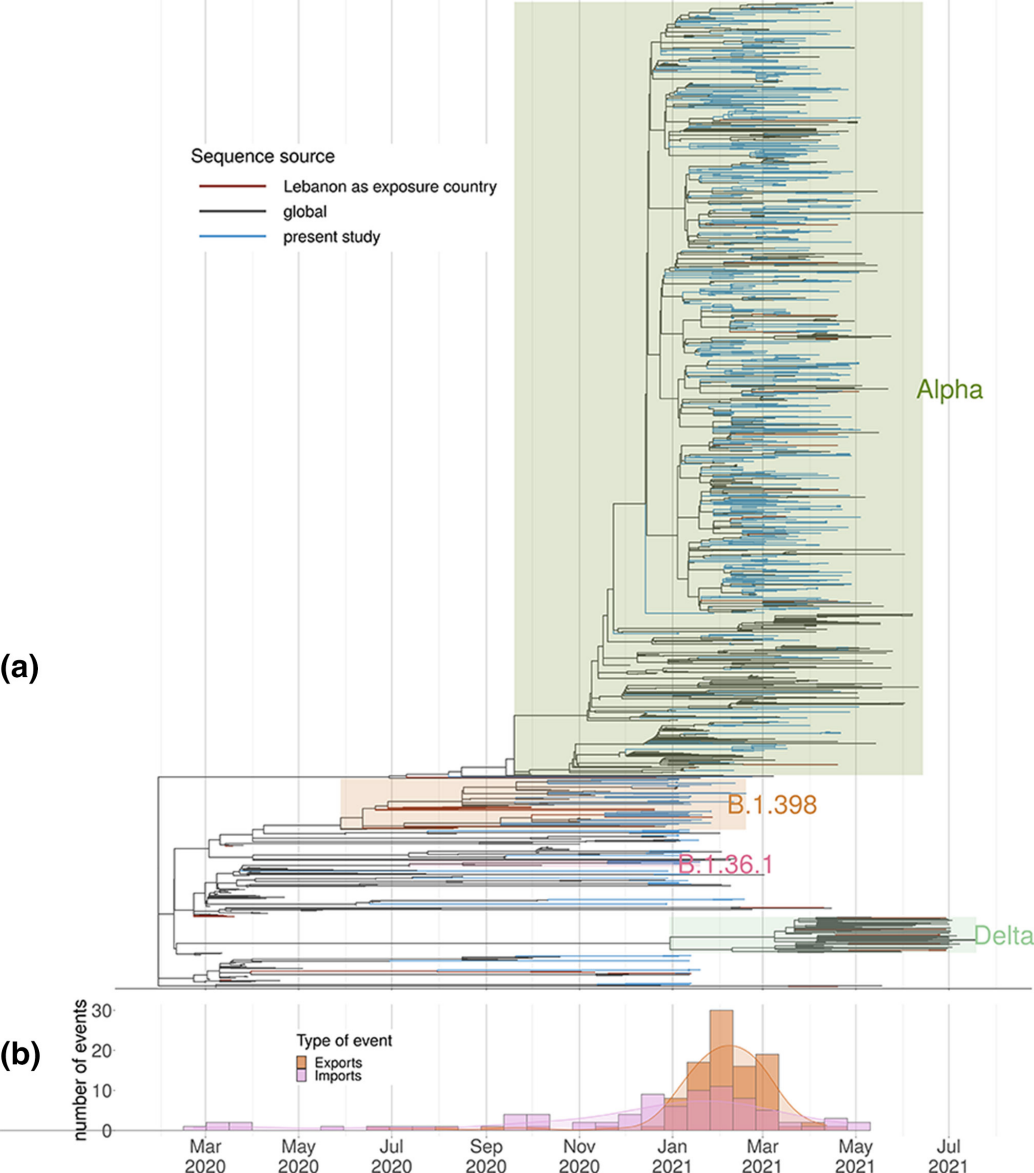
(a) Estimated divergence times using all samples with complete data information, from all lineages. (b) Estimated number of importations and exportations into/from Lebanon, based on the tree from (a).

The GISAID database houses 39 SARS-COV-2 genome sequences that were generated before this study from samples taken in Lebanon or from individuals who had recently left the country (Table 1). These include eight samples related to travel outside of Lebanon and two genomes sequenced in Japan. Analyses of these genomes reveals that at least nine SARS-CoV-2 lineages were circulating in Lebanon up to late December 2020, with no single lineage dominating (Fig. 1a).

**Table 1.** PANGO lineages of genomic data from GISAID (3 August 2021) where Lebanon is the country of exposure, together with genomes sequenced in the present study

Lineage	GISAID	This study
B.1.1.7	23	782
B.1.398	25	38
B.1	8	18
B.1.1	2	8
B.1.36.1		7
B.1.1.1		3
B.1.177.86		2
B.1.36	1	2
B.1.438	1	2
B.1.467		2
B.1.1.315	1	1
B.1.1.398		1
B.1.160		1
B.1.177		1
B.1.177.53		1
B.1.243		1
B.1.258		1
C.1		1
L.3	1	1
B.1.1.274	2	
B.1.1.487	1	
B.1.351	1	
B.1.36.35	1	
B.1.525	1	
B.1.617.2	8	
B.1.627	1	

**Fig. 3.**
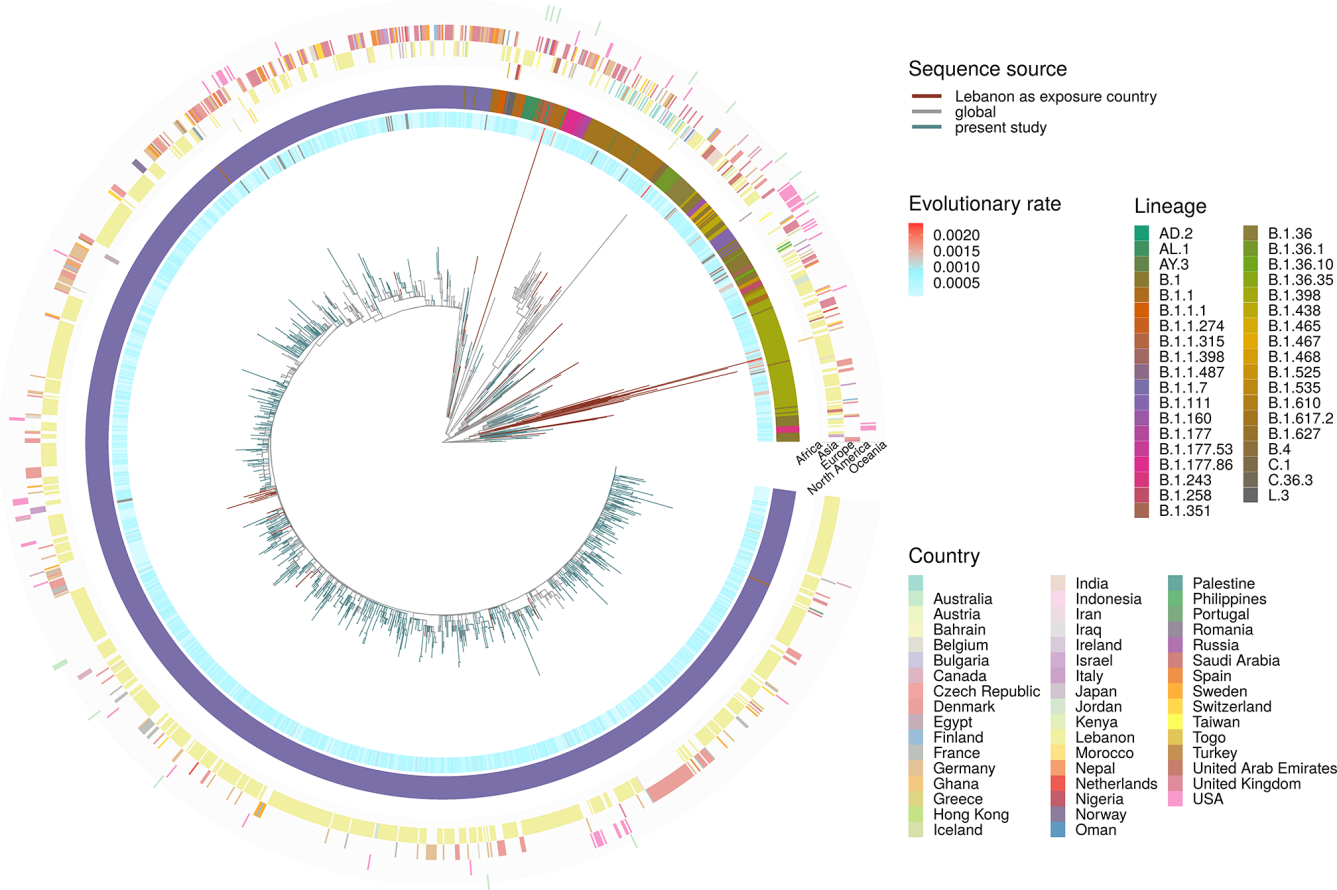
Phylogeny of selected lineages including genomes from Lebanon. Samples sequenced for the current study (blue), GISAID sequences where Lebanon is identified as the source of exposure (brown) and with a few closely related sequences from abroad. The apparent evolutionary rate in substitutions per site per year, from a timetree relaxed clock analysis, is shown in the innermost ring indicating sequences deviating from a molecular clock in red. The middle ring shows the PANGO lineages, and the outer ring describes the countries of exposure, partitioned into continents (five columns) to facilitate visualization.

**Fig. 2.**
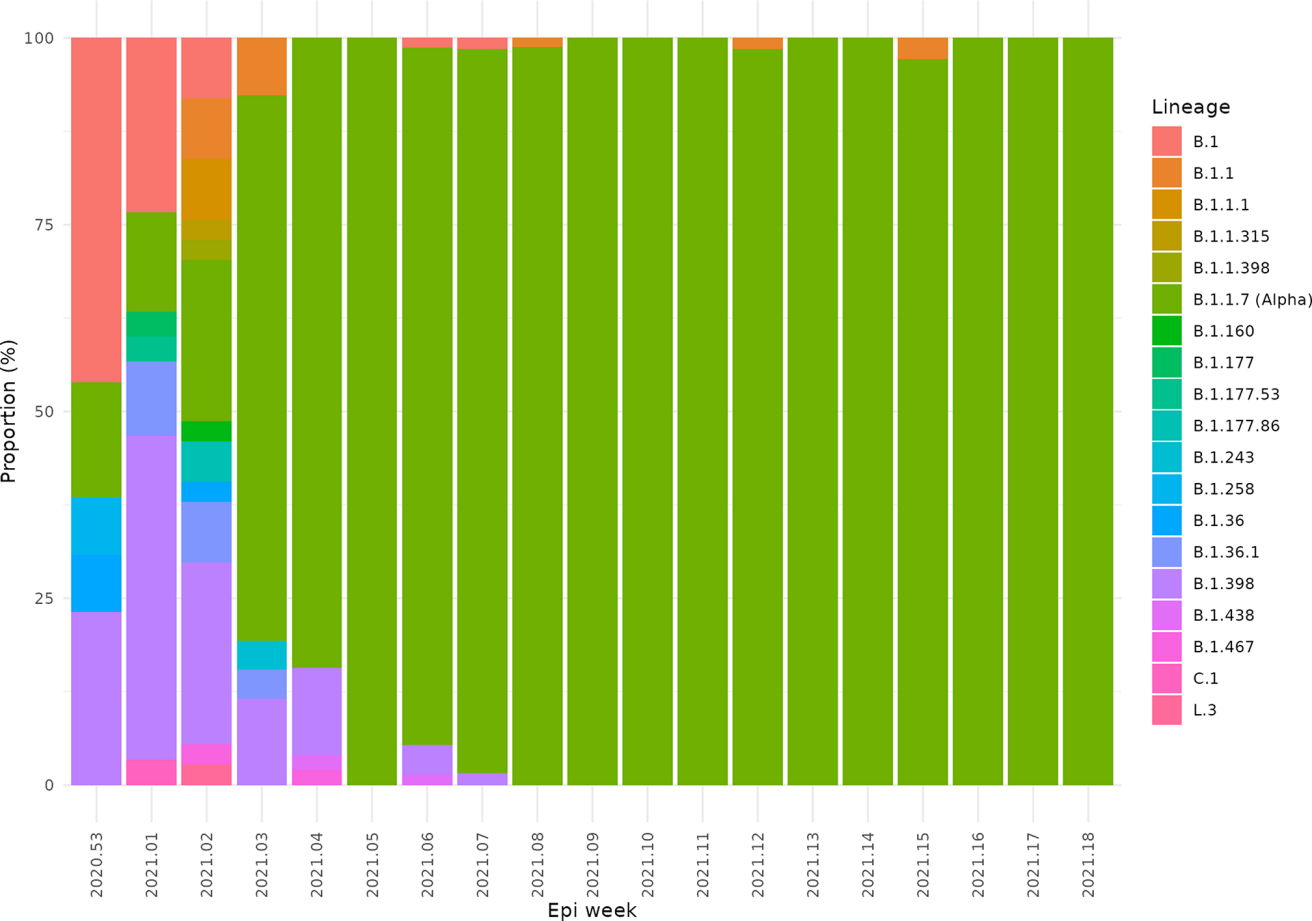
Change in the proportions of PANGO lineages between January and May 2021 by week in Lebanon showing the emergence and dominance of the Alpha variant.

The Alpha variant was first detected in Lebanon on 12 December 2020 in a traveller from the UK and Alpha is present in the community samples sequenced as part of this study on 22 December 2020. By the second week in January 2021, the Alpha variant was present in five of Lebanon’s eight provinces, and by the end of that month had been detected in seven provinces (Table S1), with the apparent absence in Akkar best explained by undersampling. The Alpha variant remained the only VOC detected via community sequencing in Lebanon up until June 2021 (Fig. 2).

There were no discernible links to sex, age (Fig. S2), or nationality (Fig. S3) during establishment of the Alpha variant. Maximum-likelihood evolutionary analysis revealed some evidence of local clustering, which is to be expected given the small size of the country (Figs 1 and S1). Phylogenetic analysis of Alpha-variant genomes revealed considerable diversity, suggesting over 40 independent importations (Figs 1 and 3b), although this is likely to be a substantial underestimate [33].

**Fig. 4.**
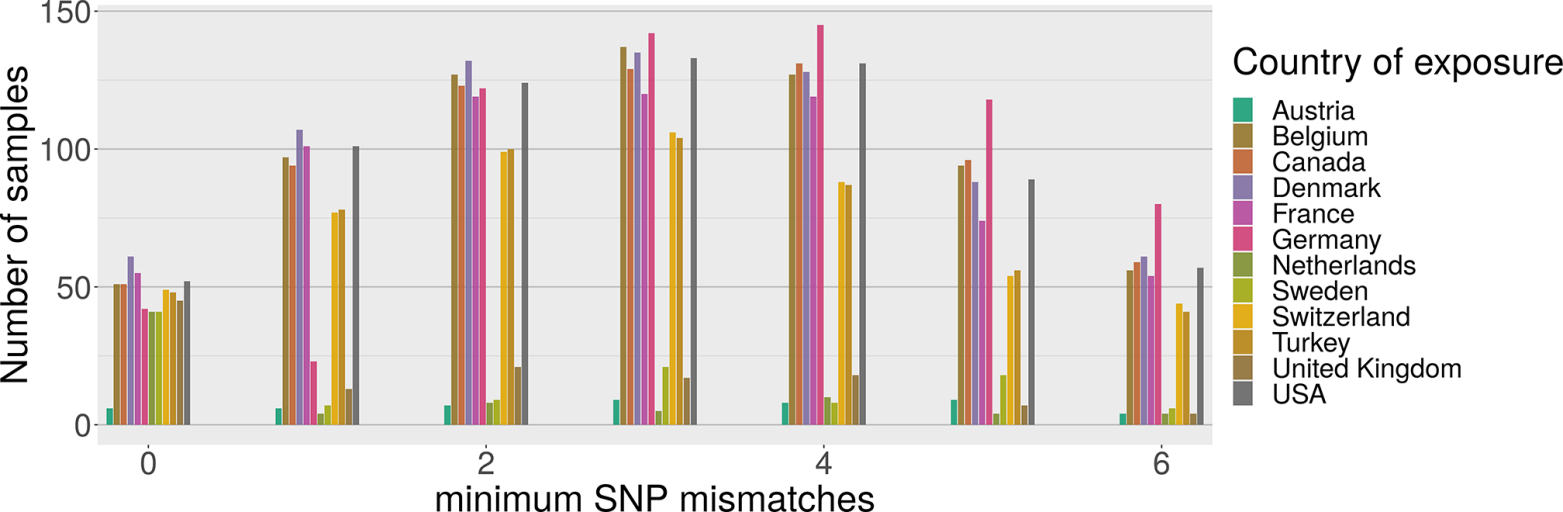
Distribution of best matches per country between local Alpha variant samples and global sequences from GISAID (27 July 2021). For each sample we find the 128 closest samples in GISAID and keep track of the best match per country. Only the most common countries are shown above, and the figure is truncated at six mismatches.

Comparisons with sequences in GISAID (see Supplementary Material 3) revealed that genomes identical to an Alpha variant from Lebanon were found in 25 countries (Fig. 4). However, due to the slow mutation rate of SARS-CoV-2 and large variation in incubation time, it is not possible to unambiguously identify the transmission route from genomic data alone. Genomes from 11 countries have a perfect match to at least 40 Lebanese genomes, with several European countries providing a larger number of matches than the UK, despite the UK providing over 41 % of the genomes from Europe during the study period and the location where the Alpha variant was first detected. This suggests that seeding of the Alpha variant into Lebanon probably occurred most often via intermediate countries rather than directly from the UK.

The terminal substitution rates using a relaxed molecular clock model were estimated at 7.6×10^−4^ (±2.2×10^−4^) substitutions per site per year for the Alpha samples sequenced as part of this study, against 6.2×10^−4^ (±2.8×10^−4^) substitutions per site per year for the global Alpha sequences included in the present analysis. This is in agreement with other estimates, which show no noticeable differences in rates except for episodic events leading to new lineages [34]. The overall estimated rates may also be influenced by the analysis time span and model choice, which explains subtle differences between publications [35]. The same behaviour was observed for the other lineages included in the present study, with no discernible differences in the rates between countries.

### Replacement with Delta

No genome of samples collected in Lebanon are available for a 7 week period from 5 May to 26 June 2021, which corresponded to a period of very low case numbers. The first cases of Delta in The Gambia were detected in travellers from Lebanon (accession numbers: EPI_ISL_2820697, EPI_ISL_2820699) by genome sequencing at the MRC Unit The Gambia at the end of May 2021, indicating community transmission of Delta was already occurring in Lebanon. In that time there was a complete replacement of Alpha with Delta. A total of 28 samples were sequenced by the Lebanese American University (LAU) between 25 June 2021 and 3 July 2021, 100 % of which were Delta (Table S2). This rapid replacement of the dominant variant reflects the experience of many other countries worldwide, such as the UK, Denmark and the USA [1].

### Low-frequency mutation analysis

The mutations of 4119970 genomes were analysed to place the low-frequency mutations found in the samples sequenced within this study into the global context of the major VOCs. Full details of each mutation are given in Table S3, with the frequency of the Lebanese mutations compared to their prevelance in the global dataset, excluding Lebanese genomes, and then compared to each major VOC: Alpha (519295 genomes), Delta (2244642 genomes) and Omicron (771548 genomes). Taking mutations which occur in fewer than 50 samples from this study, there were five that were present in over 90 % of Omicron genomes. On further examination, a single amino acid deletion at 21995–21997 was found to be different to the 3 aa deletion (9 nt) in Omicron (BA.1), but it does indicate that this region of the Spike gene is under pressure. Mutations G23048A (S:G496S), found only in BA.1 genomes, G22992A (S:S477N), C26270T (E: T9I) and C25000T (non-synonymous) found in nearly all Omicron genomes represented the remainder of the variants seen. These also independently arose sporadically in Alpha and Delta globally.

## Discussion

This study details a ten-fold increase in the number of available SARS-CoV-2 genomes for Lebanon, providing insights into the dynamics of the pandemic. The first COVID-19 cases in Lebanon in February 2020 were dominated by lineage B.1. Phylogenetic analysis of whole genome sequences in a previous report [36] and in this study confirmed multiple introductions via international travel, followed by community spread that led to strict lockdown measures. Genome sequencing facilities in low- and middle-income countries remain scarce and are often associated with limited resources. In Lebanon, at the time of writing, 1052 (905 from this study) SARS-CoV-2 genomes have been deposited on GISAID, representing two sequenced genomes per 1000 cases. Genome sequencing and phylogenetic analysis are key strategies to investigate and track the circulating viral lineages, identify routes of transmissions, and monitor and mitigate the spread of early introductions of VOCs.

Up to December 2020, no single variant completely dominated in Lebanon, although the B.1.398 lineage was a constant feature. This was in stark contrast to other countries which saw wholesale replacements of the wild-type with a single lineage. In Europe, after dispersal from Spain in summer 2020, the B.1.177 lineage had become the dominant lineage by the autumn of that year, probably reflecting the fitness advantage provided by the A222V mutation in the spike protein [37].

The first detected case of the Alpha variant was on 12 December 2020 in a traveller from the UK. During the holiday period of December 2020, lockdown restrictions were eased, followed by an increase in reported COVID-19 cases, which reached 1843 per day by 31 December 2020 [13]. The country went into a nationwide lockdown on 7 January 2021. In this study we found that Alpha was present in the community on the earliest collection date (29 December 2020) and 3 weeks later cases could be detected in five out of eight provinces, with Alpha accounting for 84 % of cases (*n*=43/51) by the last week of January 2021.

Following the emergence of the Alpha variant in the UK, additional restrictions were applied to travellers arriving from the UK in the Lebanon. Nonetheless, the Alpha variant swept rapidly through Lebanon causing the largest wave seen up to that point, with cases peaking in January 2021. Directionality is difficult to establish using genome sequencing alone. However, our sequence comparison analysis (Fig. 4) shows that travel from the UK was probably not the primary driver for importation of the Alpha variant into Lebanon given that Alpha variant genomes from 11 countries provide perfect matches to genomes from Lebanon. These best matches come not from the UK, but instead from Belgium, Denmark, France, Germany and Switzerland, as well as Turkey, Canada and the USA. These countries have large Lebanese diaspora populations.

The earliest detection of the Alpha variant in a traveller from Lebanon occurred in Singapore with a testing date of 16 December 2021. This suggests that the Alpha variant was already established in Lebanon at this point. The Alpha variant spread rapidly to every province in Lebanon. There is evidence of distinct clusters of Alpha establishing within particular provinces within Lebanon with onward seeding of other provinces. Exports from Lebanon to other countries were estimated to peak at the start of February (Fig. 1b). Public health measures which appeared to suppress transmission of earlier lineages of SARS-CoV-2 were insufficient to control the Alpha variant, which showed increased transmissibility [7]. This highlights the need for rapid prospective genomic surveillance to help inform public health measures.

No other VOCs were observed during this period, up to the beginning of May 2021. Delta was first identified in India and rapidly became the dominant variant in the UK in April 2021. The first indication that the Delta variant had reached Lebanon was in cases from the Gambia with a travel history from Lebanon, which represented the first identified cases of that variant in the Gambia. This suggests that by mid-May 2021, Delta was already present in the community in Lebanon, even though overall case numbers were low. Genomic surveillance started in Lebanon at the end of June 2021 and 100 % of community cases were identified as Delta, confirming that the Delta variant is now widespread in the community. The importation of Delta and resulting community transmission probably occurred silently many weeks before the official announcement of its discovery on 2 July 2021. This highlights the need for ongoing rapid prospective genome sequencing of community cases in addition to testing at borders.

An analysis of mutations found at low proportions in Lebanese genomes, which were subsequently found in global Omicron genomes at high proportions, was undertaken. One mutation (S:S477N) independently arose in a Lebanese Alpha genome that had been previously observed in summer 2020 in the expansion of B.1.177, probably emerging from Spain [37]. The repeated observation of this mutation in successful lineages indicates it confers a fitness advantage. It is important to monitor low-frequency mutations as they can give insights into potential future fitness-giving mutations.

## Conclusion

Our results reveal how a variant could rapidly become dominant and that travel restrictions alone may be insufficient to prevent transmission. We have highlighted the importance of sequence-based surveillance in monitoring SARS-CoV-2 community transmission, identifying and detecting emerging variants. Targeted travel restrictions did appear to be effective. Integrated genome sequencing plays a critical role in guiding interventions, containment strategies and travel restrictions. Through support of WHO, the recently launched national genomic surveillance initiative should be further expanded and supported to track the emergence, introduction and spread of new SARS-CoV-2 variants.

## Supplementary Data

Supplementary material 1

Supplementary material 2

Supplementary material 3

Supplementary material 4
