# Supplementary material for: Replacement of the Alpha variant of SARS-CoV-2 by the Delta variant in Lebanon between April and June 2021
Source: Microb Genom. 2022 Jul 25;8(7):mgen000838. doi: 10.1099/mgen.0.000838 (PMC9455693; doi:10.1099/mgen.0.000838)
Supplement: Supplementary material 1 [file mgen-8-838-s001.pdf]

Supplementary

Supplementary Table 1: Spreadsheet of sample metadata and accession numbers.
Supplementary Table 2: GISAID acknowledgements.
Supplementary Table 3: Spreadsheet of mutations in this study, placed in the context of variants of concern globally.

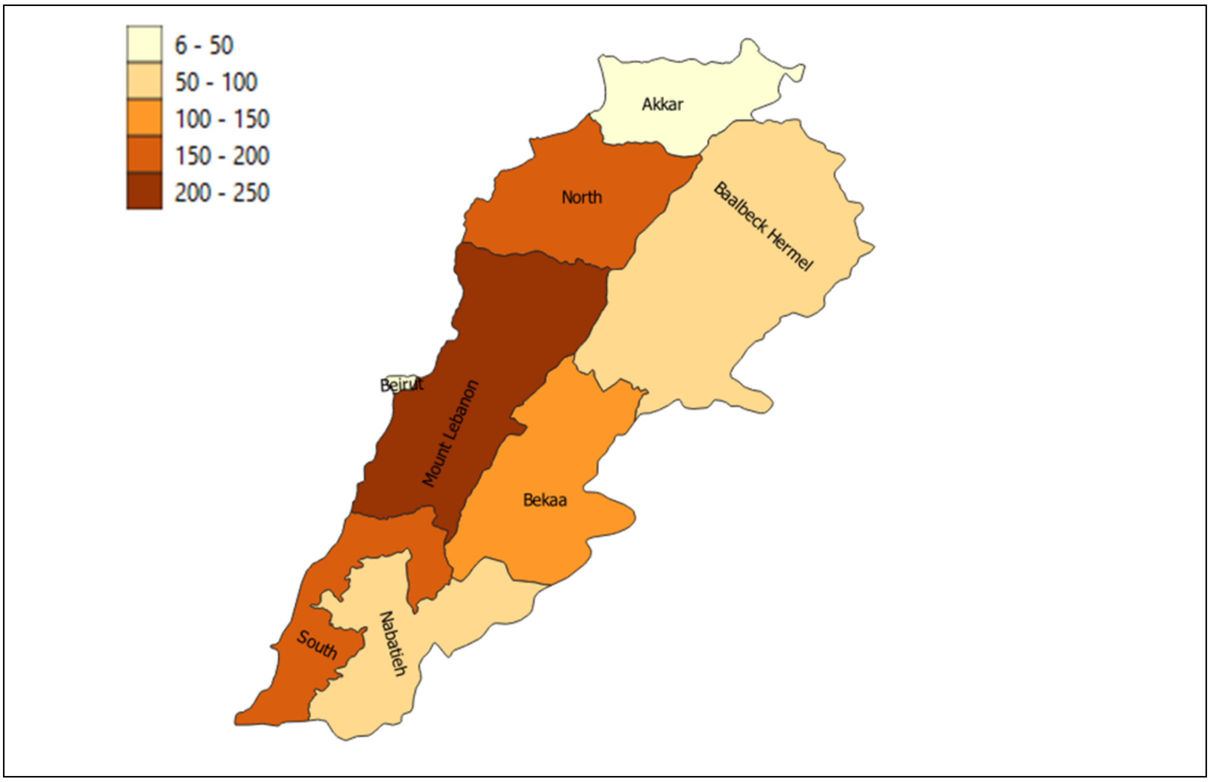

Supplementary Figure 1: Distribution of sequenced cases in this study for each province.

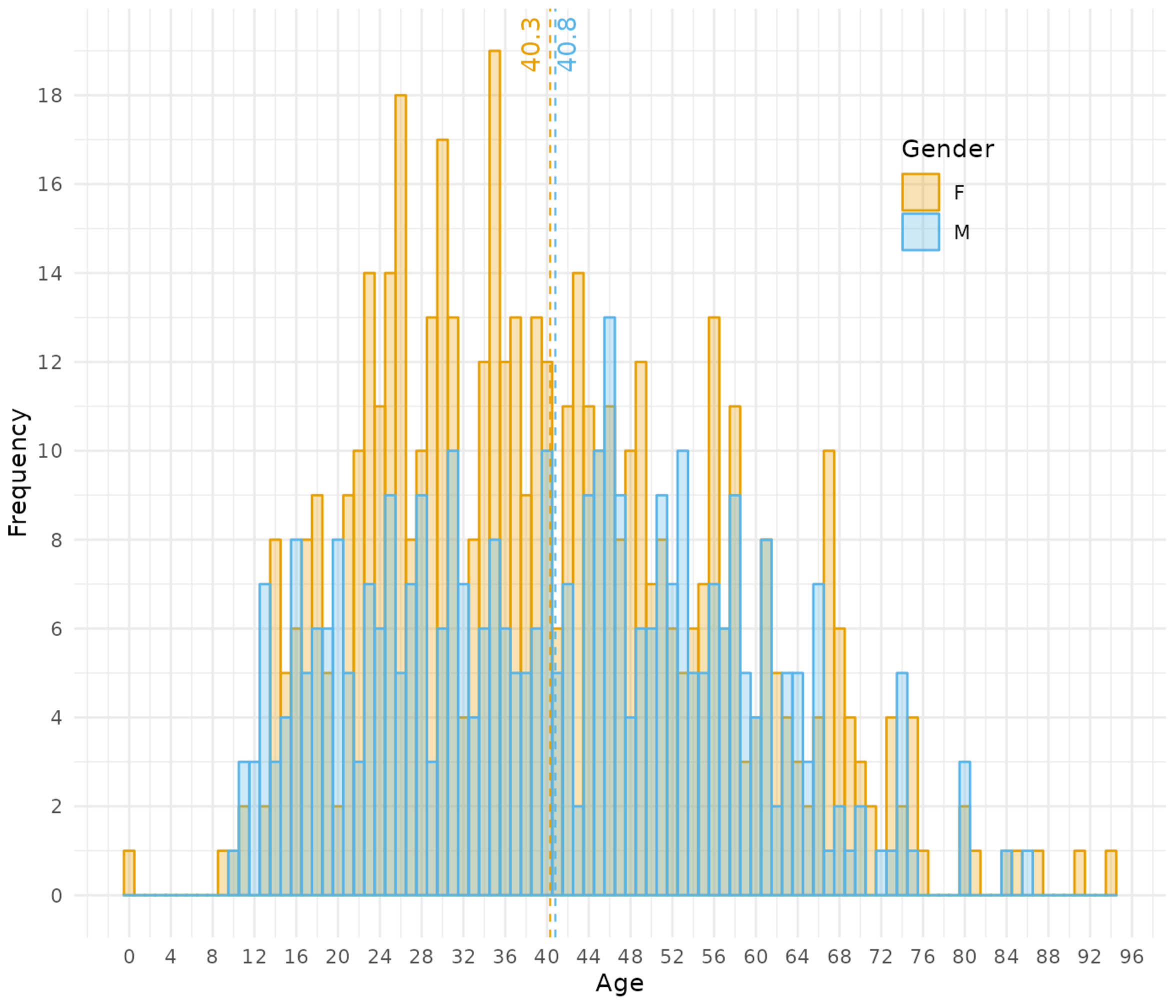

Supplementary Figure 2: Demographics of samples from cases included in this study. Two cases with unknown sex were excluded. An age of zero corresponds to a baby under the age of 1. The mean age of the female cases was 40.3 years (± 16.5 SD) and the male cases was 40.8 years (± 16.8 SD).

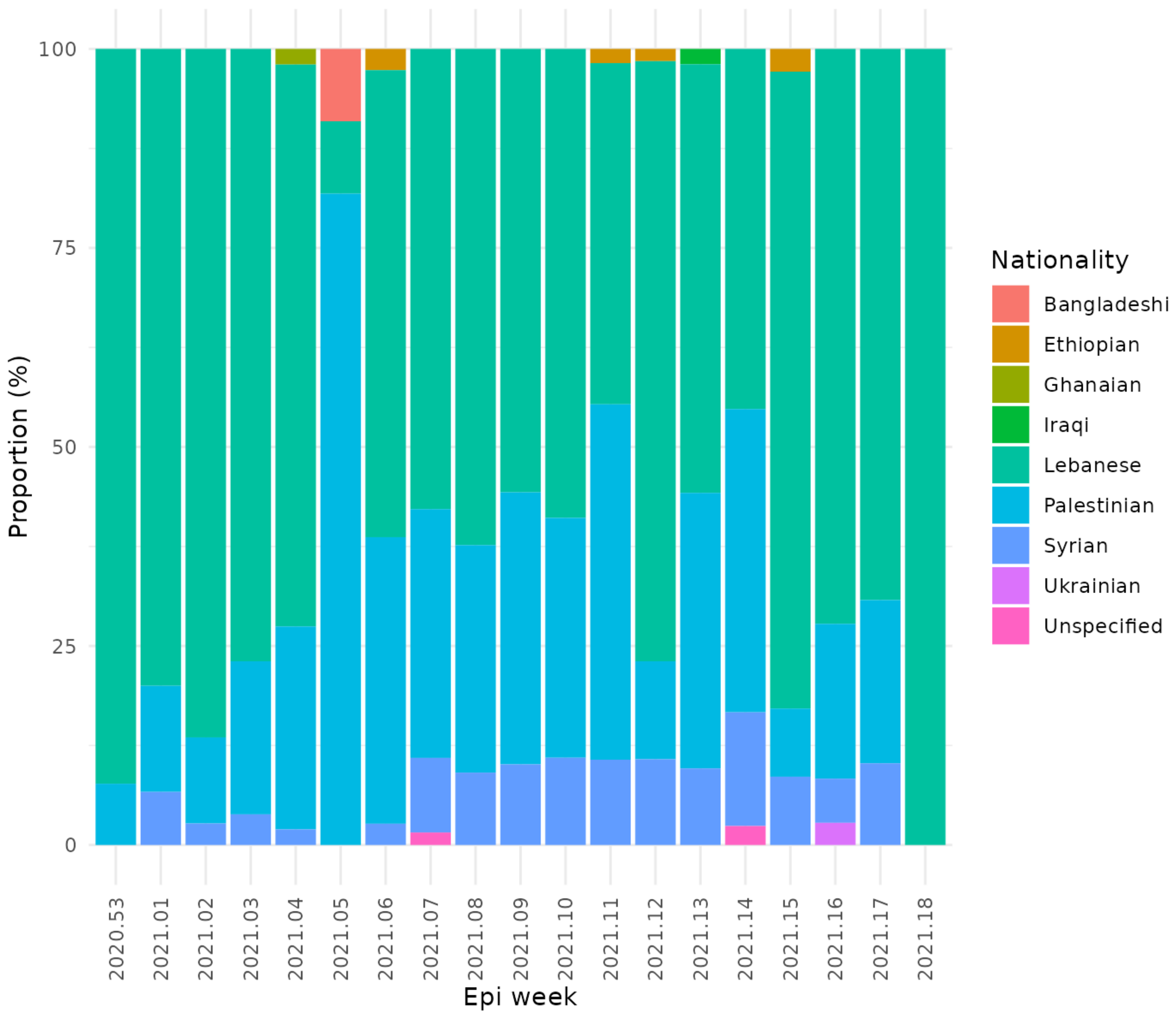

Supplementary Figure 3: The proportion of self-declared nationalities of cases by week from January to May 2021.
